# RSPO3 impairs barrier function of human vascular endothelial monolayers and synergizes with pro-inflammatory IL-1

**DOI:** 10.1186/s10020-018-0048-z

**Published:** 2018-08-29

**Authors:** Tom Skaria, Esther Bachli, Gabriele Schoedon

**Affiliations:** 1 Inflammation Research Unit, Division of Internal Medicine, University Hospital Zürich, Rämistrasse 100, CH-8091 Zürich, Switzerland; 2 Department of Medicine, Uster Hospital, Brunnenstrasse 42, CH-8610 Uster, Switzerland

**Keywords:** Endothelial dysfunction, RSPO3, Inflammation, Vascular leakage, IL-1β

## Abstract

**Background:**

Endothelial barrier dysfunction characterized by hyperpermeability of the vascular endothelium is a key factor in the pathogenesis of chronic inflammatory diseases and affects clinical outcomes. In states of chronic inflammation, mediators secreted by activated immune cells or vascular endothelium may affect the barrier function and permeability of the vascular endothelium. The matricellular R-spondin family member RSPO3 is produced by inflammatory-activated human monocytes and vascular endothelial cells, but its effects in the regulation of vascular endothelial barrier function remains elusive.

**Methods:**

The present study investigates the effects of RSPO3 on the barrier function of adult human primary macro- and micro- vascular endothelial monolayers. Tight monolayers of primary endothelial cells from human coronary and pulmonary arteries, and cardiac, brain, and dermal microvascular beds were treated with RSPO3 either alone or in combination with pro-inflammatory mediator IL-1β. Endothelial barrier function was assessed non-invasively in real-time using Electric Cell-substrate Impedance Sensing.

**Results:**

RSPO3 treatment critically affected barrier function by enhancing the permeability of all vascular endothelial monolayers investigated. To confer hyperpermeable phenotype in vascular endothelial monolayers, RSPO3 induced inter-endothelial gap formation by disrupting the β-catenin and VE-cadherin alignment at adherens junctions. RSPO3 synergistically enhanced the barrier impairing properties of the pro-inflammatory mediator IL-1β.

**Conclusion:**

Here, we show that the matricellular protein RSPO3 is a mediator of endothelial hyperpermeability that can act in synergy with the inflammatory mediator IL-1β. This finding stimulates further studies to delineate the endothelial barrier impairing properties of RSPO3 and its synergistic interaction with IL-1β in chronic inflammatory diseases.

**Electronic supplementary material:**

The online version of this article (10.1186/s10020-018-0048-z) contains supplementary material, which is available to authorized users.

## Background

The semipermeable endothelial barrier plays an important role in maintaining vascular homeostasis as the tightly controlled vascular permeability permits the macromolecule transport, immune surveillance and fibrin barrier deposition at inflamed sites (Weis, 2008). Hyperpermeability of the endothelial barrier causing vascular leakage of blood components and immune cell transmigration is a crucial factor in the pathogenesis of acute and chronic inflammatory diseases, stroke, and tumor inflammation. Strategies aimed at stabilizing endothelial barrier homeostasis prevent tissue damage and have been suggested to improve therapeutic outcomes in inflammatory diseases (Chava et al., 2012; Han et al., 2013).

R-spondin (RSPO)-3, a member of the RSPO family of matricellular signaling proteins involved in vascular homeostasis (Aoki et al., 2007; Knight & Hankenson, 2014; Scholz et al., 2016) promotes oncogenesis in solid tumors (Chartier et al., 2016; Marcucci et al., 2016; Picco et al., 2017) and hematologic malignancies (van Andel et al., 2017; Wang et al., 2013). RSPO act as enhancers of signaling pathways mediated by Wnt ligands (Chartier et al., 2016; Knight & Hankenson, 2014; van Andel et al., 2017), the latter consists of 19 secreted lipid modified proteins with critical roles in regulating cell adhesion and endothelial barrier function (Lim et al., 2017; Skaria et al., 2017a). Previous studies showed that the prototypic pro-inflammatory mediator IL-1β (Dinarello, 2011) significantly induces RSPO3 expression in human macrophages (Williams et al., 2009) and vascular endothelial cells (accession GSE62281, NCBI GEO data repository) (Skaria et al., 2017a). However, the role of RSPO3 in the regulation of vascular endothelial barrier function remains still unclear. Here, we investigated the effect of RSPO3 on endothelial monolayer barrier function in real-time using electric cell-substrate impedance sensing (ECIS). This study shows for the first time that RSPO3 (1) directly and dose-dependently enhances the permeability of human primary endothelial cell monolayers independent of their anatomical origin, and (2) co-operates with IL-1β, further enhancing IL-1β-induced permeability of macro-and micro- vascular endothelial monolayers.

## Methods

### Cell culture

Human coronary artery endothelial cells (HCAEC), human pulmonary artery endothelial cells (HPAEC), human cardiac microvascular endothelial cells (HCMVEC), human dermal microvascular endothelial cells (HDMVEC, all from Clonetics, Lonza), and human brain microvascular endothelial cells (HBMVEC, Cell Systems) were propagated as described (Skaria et al., 2017a) and treated with recombinant human RSPO3 (doses ranging from 250 to 1000 ng/mL, PeproTech) and recombinant human IL-1β (20 U/mL, PeproTech) as given in the supplementary methods (Additional file 1: Supplementary methods ). For details on endothelial cell characterization, see supplementary methods.

### Immunoblotting

Immunoblotting of RSPO3 and quantification of band densities were carried out as described (Skaria et al., 2017b), and given in the supplementary methods (Additional file 1). The following primary antibody was used with dilution indicated: rabbit anti-RSPO3, polyclonal (1:500, Abcam). Anti-rabbit IgG-HRP-linked whole antibody, from donkey (1:5000, GE Healthcare UK Limited) was used as the secondary antibody.

### Live recording of endothelial monolayer barrier function using ECIS

The response of endothelial monolayer barrier to a particular stimulus can be assessed in real-time in a fully standardized manner by continuously recording changes in trans-endothelial electrical resistance (TEER) using ECIS (Bernas et al., 2010). Endothelial barrier function was continuously recorded using the 8W10E+ electrode chamber arrays and ECIS Z-Theta system (Applied Biophysics) with associated software v.1.2.126 PC, as described (Skaria et al., 2017a) and given in the supplementary methods (Additional file 1: Supplementary methods).

### Immunofluorescence staining

Immunofluorescence staining of β-catenin and VE-cadherin were performed as described (Skaria et al., 2016) and given in the supplementary methods (Additional file 1). Quantitative assessment of gap index and gap size index were carried out as described (Fraccaroli et al., 2015) and given in the supplementary methods (Additional file 1: Supplementary methods).

### Statistical analysis

Data were analyzed using GraphPad Prism software version 5.04 (GraphPad Software, San Diego, CA). An unpaired 2-tailed Student’s *t*-test or, for comparing data among groups, ANOVA followed by Bonferroni post hoc test was used and differences were considered statistically significant at *p* < 0.05.

## Results and discussion

### RSPO3 enhances the permeability of vascular endothelial monolayers

**Fig. 1:**
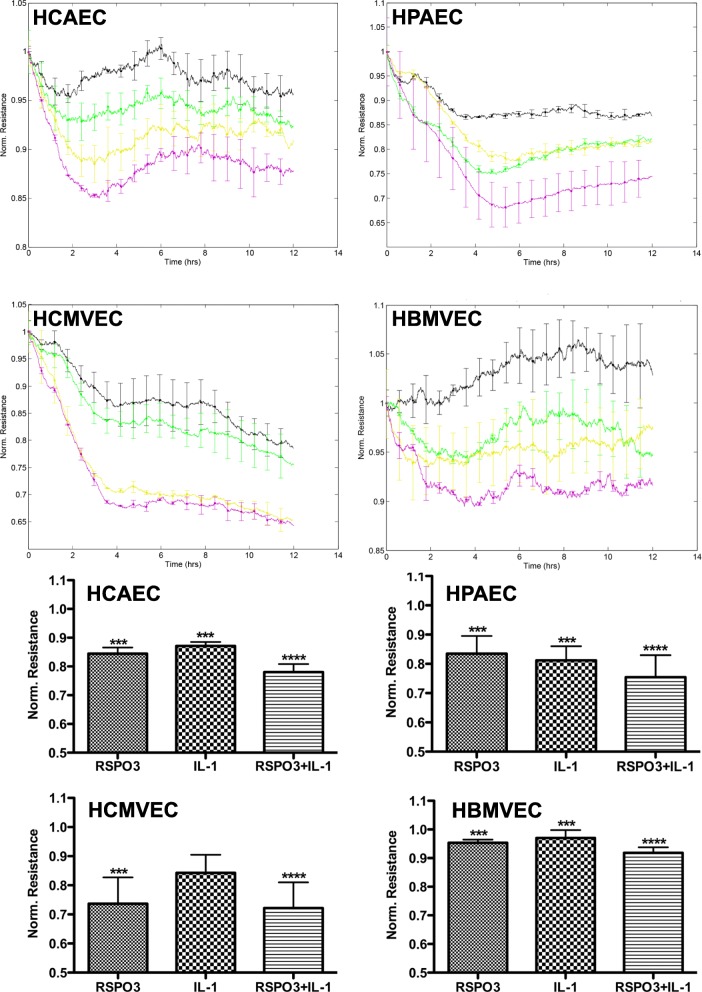
Automated real-time assessment of endothelial monolayer barrier function using the ECIS system. Uniform tight monolayers of HCAEC, HPAEC, HCMVEC and HBMVEC cultured in stabilized and collagen coated 8W10E+ ECIS culture chambers were treated with RSPO3 (500 ng/mL), IL-1β (20 U/mL) or a combination of RSPO3 and IL-1β. The line graphs are the original ECIS measurements obtained using ECIS Z Theta system equipped with v.1.2.126 PC software and from one representative of three independent experiments run in duplicates at 4000 Hz (indicative of cell-cell adhesion tightness). Bar graphs show the data of barrier function measurements continuously recorded for and at 12 h from three independent experiments run in duplicates. Black, vehicle; Green, IL-1β; Yellow, RSPO3; Purple, RSPO3 + IL-1β. Error bars are mean ± S.D. ***P < 0.001 versus vehicle treatment, ****P < 0.001 versus RSPO3 and IL-1β single treatments

**Fig. 2:**
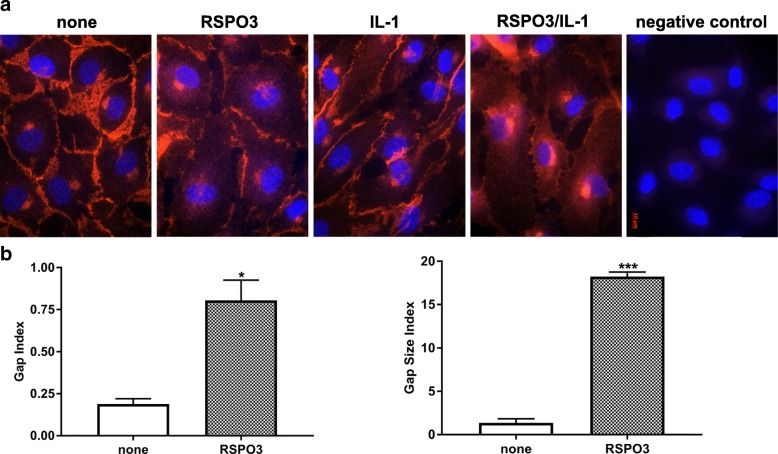
Inter-cellular gaps in vascular endothelial monolayers treated with RSPO3 or IL-1β. **a** Immunofluorescence staining of VE-cadherin (red) and DAPI staining of nuclei (blue) in HCAEC either untreated, or treated with RSPO3, IL-1β and combined IL-1β and RSPO3 for 6 h. Microphotographs were taken using a Zeiss Axioskope equipped with Axio-CamMRm and AxioVision Rel.4.6 software and are representative of three independent experiments run in triplicates. Original magnification, 630×. **b** Quantification of inter-endothelial gap index and gap size index in HCAEC treated with RSPO3. Data are mean ± S.E.M of three independent experiments run in triplicates, *P < 0.05, ***P < 0.001 versus untreated (none)

To functionally test the effect of RSPO3 on monolayer permeability, human primary macro- (HCAEC, HPAEC) and micro- (HCMVEC, HBMVEC, HDMVEC) vascular endothelial cells grown to tight monolayers in 8W10E+ array slides (Additional file 2: Figure S1A, B) were treated with RSPO3 as indicated (Additional file 1: supplementary methods, Additional file 3: Figure S2) and changes in TEER of the endothelial monolayers were continuously recorded for 12 h. As indicated by the significant decrease in TEER, RSPO3 treatment dose-dependently (Additional file 3: Figure S2) and at an optimal dose of 500 ng/mL enhanced the permeability in all endothelial monolayers tested independent of the anatomical type. RSPO3-induced alterations in TEER became significantly evident 2 h after beginning of treatment and persisted for more than 6 h (Fig. 1, Additional file 4: Figure S3). Since endothelial hyperpermeability occurs due to increased actin cytoskeleton polymerization and, in consequence gap formation caused by β-catenin and VE-cadherin disruption at inter-endothelial junctions (Vandenbroucke et al., 2008; Weis, 2008), we next checked if RSPO3 alters the actin cytoskeleton and generates inter-endothelial gaps. In non-treated cells, thin actin filaments were scattered and located at the cellular periphery as cortical actin (Additional file 5: Figure S4), and β-catenin (Additional file 6: Figure S5) and VE-cadherin (Fig. 2) were assembled at the cellular periphery forming tight boarders between inter-cellular membranes. Treatment with RSPO3 enhanced actin polymerization generating notably thicker actin fibers (Additional file 5: Figure S4), and disrupted β-catenin (Additional file 6: Figure S5) and VE-cadherin at inter-cellular boarders, forming large inter-endothelial gaps (Fig. 2a, b).

### RSPO3 synergistically enhances the barrier destructive effects of the pro-inflammatory mediator IL-1β

IL-1β secreted by activated leucocytes critically mediates pro-inflammatory responses in acute and chronic inflammatory diseases, cardiovascular diseases and tumor inflammation (Dinarello, 2011). Because RSPO3 expression is induced by IL-1β in human leucocytes (Williams et al., 2009) and vascular endothelium (Skaria et al., 2017a) (Additional file 7: Figure S6), and can be simultaneously present together with IL-1β during the inflammatory response, we investigated the effect of combined IL-1β/RSPO3 on vascular endothelial barrier function. IL-1β treatment alone significantly increased permeability in HCAEC, HPAEC, HBMVEC (Fig. 1), and HDMVEC monolayers (Additional file 4: Figure S3). Combined IL-1β/RSPO3 treatment synergistically increased permeability in HCAEC, HPAEC, HCMVEC, HBMVEC (Fig. 1) and HDMVEC monolayers (Additional file 4: Figure S3). The synergistic effect of RSPO3 with IL-1 was more pronounced in HCAEC, HPAEC, HBMVEC (Fig. 1) and HDMVEC (Additional file 4: Figure S3) monolayers compared to that observed in HCMVEC (Fig. 1).

Although previous studies found that activated leucocytes and vascular endothelial cells express high levels of RSPO3 (Skaria et al., 2017a; Williams et al., 2009), and its increased expression mediates pathogenesis in intestinal inflammation (Kang et al., 2016) and oncogenic pathways in cancers such as colon, lung (Chartier et al., 2016; Marcucci et al., 2016; Picco et al., 2017) and multiple myeloma (van Andel et al., 2017), its function in the vascular system remains still elusive. Most of the studies investigating adult human vascular endothelial function in vitro rely solely on cultured human umbilical vein endothelial cells (HUVEC). It was later reported that HUVEC obtained from the immune naïve fetal tissue shows significant variations in function compared with adult human vascular endothelium and hence may represent an inappropriate primary cell model of vascular endothelium (Hwang et al., 2018; O'Donnell et al., 2000; Tan et al., 2004). Therefore, to investigate the effects of RSPO3 on adult human primary macro- and micro- vascular endothelial monolayer barrier function, we employed primary endothelial cells derived from adult human coronary and pulmonary arteries, and cardiac, brain, and dermal microvascular beds, that were positively tested for vascular endothelial markers and function, and are well established as immunocompetent (Burton et al., 2011; Chandrasekar et al., 2004; Franscini et al., 2004; Quinlan et al., 1999; Skaria et al., 2017a; Skaria et al., 2016; Zeuke et al., 2002). Here we show unequivocally that RSPO3 enhances permeability of adult human macro- and micro- vascular endothelial monolayers. RSPO3, like other secreted RSPO family members are Wnt signaling enhancers functioning through Leu-rich repeat-containing G protein-coupled receptors (LGR) or syndecan-4 (SDC4) receptor to activate Wnt/β-catenin dependent or β-catenin independent Wnt/planar cell polarity (PCP) signaling respectively (Chartier et al., 2016; Knight & Hankenson, 2014; van Andel et al., 2017). The whole genome microarray profiling of 4 h and 8 h IL-1-activated human vascular endothelium (accession GSE62281, NCBI GEO data repository) in our previous study shows an upregulated expression of SDC4 along with RSPO3 (Skaria et al., 2017a). However, whether RSPO3-induced vascular endothelial barrier dysfunction is mediated through enhancement of constitutively expressed endothelial barrier impairing Wnt5A (Skaria et al., 2017a), or any other member of the Wnt family of 19 secreted signaling proteins remains unclear. It is also not clear whether a β-catenin dependent or independent Wnt signaling is involved downstream of RSPO3 in human vascular endothelium of different anatomical sites.

Endothelial barrier breakdown and in consequence, monolayer hyperpermeability causes tissue edema and contributes to morbidity and mortality associated with acute and chronic inflammatory diseases (Chava et al., 2012; Weis, 2008). In cancer, endothelial barrier dysfunction enhances angiogenesis and metastasis by facilitating the intravasation of tumor cells from the tumor into the blood vessel and tumor cells’ extravasation out of the blood vessel to the target tissues, respectively (Singleton, 2014; Weis, 2008). Another major finding of this study is the potency of RSPO3 to consistently enhance IL-1β-mediated hyperpermeability in human macro- and micro- vascular endothelial monolayers. It was shown that IL-1β induces the expression of RSPO3 in human macrophages and vascular endothelium (Skaria et al., 2017a; Williams et al., 2009). Pro-inflammatory responses mediated by IL-1 have critical implications in an expanding number of local or systemic acute and chronic inflammatory diseases such as sepsis, severe systemic inflammatory syndromes, inflammatory bowel disease and rheumatoid arthritis, and cancers such as colon and myeloma. In these pathological states, increased IL-1 expression strongly correlates with the disease progression and its neutralization leads to abrupt and sustained decrease in disease severity (Dinarello, 2011). In this context, the findings that RSPO3 is induced by IL-1 and significantly enhances IL-1- mediated hyperpermeability in both macro- (HCAEC, HPAEC) and two (HBMVEC, HDMVEC) out of three micro- (HCMVEC, HBMVEC, HDMVEC) vascular endothelial monolayers tested are of prominent interest. The present study, therefore, stimulates further studies to delineate the endothelial barrier impairing properties and mechanisms of RSPO3, and its synergistic interaction with IL-1 in acute and chronic inflammatory diseases and tumors.

## Conclusion

Taken together, we show in this short report that the matricellular protein RSPO3 is a novel permeability factor inducing barrier dysfunction in human primary vascular endothelial monolayers of macro- and micro- vascular origin and RSPO3 acts synergistic with the prototypic pro-inflammatory mediator IL-1β. These findings expand the knowledge of the role of RSPO3 in the human vascular system and prompt further studies to investigate whether RSPO3 causes vascular leakage in IL-1-mediated inflammatory diseases and cancers.

## Additional files

Additional file 1: Supplementary methods. (PDF 124 kb)

Additional file 2: **Figure S1.** Confluent tight endothelial monolayer formation in 8W10E+ ECIS arrays. Immediately after cell seeding, resistance measurements (in Ohms) were started and are displayed as normalized resistance (subsequent values were divided by initial values). Increase in resistance with respect to time denotes that cells were forming contacts between each other. The steady state shows the stage at which maximum resistance is reached to form a tight monolayer. Resistance measurements were carried out in duplicate wells which were grouped and averaged to plot as single curve. Error bars represent mean ± S.E.M. Figures show the original plot of resistance measured at 4000 Hz (indicative of cell-cell adhesion tightness). Red, blue: record of monolayer formation of untreated (A) HCAEC and (B) HCMVEC grown in duplicate wells from two independent experiments. All treatments for subsequent assessment of barrier function were started after formation of stable tight monolayers. (PDF 636 kb)

Additional file 3: **Figure S2.** Dose response assessment of RSPO3 on endothelial barrier function using the ECIS system. Uniform tight monolayers of HCAEC cultured in stabilized and collagen coated 8W10E+ ECIS array chambers were treated with 250 ng/mL, 500 ng/mL and 1000 ng/mL RSPO3. Data shown are the original resistance measurements conducted at 4000 Hz (indicative of cell-cell adhesion tightness) and are representative of three independent experiments. Black, vehicle; Purple, RSPO3 (250 ng/mL); Yellow, RSPO3 (500 ng/mL); Grey, RSPO3 (1000 ng/mL). (PDF 304 kb)

Additional file 4: **Figure S3.** Assessment of barrier function in dermal microvascular endothelial monolayers. Uniform tight monolayers of HDMVEC cultured in stabilized and collagen coated 8W10E+ array chambers were treated with RSPO3, IL-1β or a combination of RSPO3 and IL-1β. (A) Original ECIS plot of resistance measurements (line graph) from one representative of three independent experiments run in duplicates at 4000 Hz (indicative of cell-cell adhesion tightness). (B) Bar graph showing the data of barrier function measurements continuously recorded for and at 12 h from three independent experiments run in duplicates. Black, vehicle; Green, IL-1β; Yellow, RSPO3; Purple, RSPO3 + IL-1β. Error bars are mean ± S.D. ***P < 0.001 versus vehicle treatment, ****P < 0.001 versus RSPO3 and IL-1β single treatments. (PDF 242 kb)

Additional file 5: **Figure S4.** Actin cytoskeletal changes in vascular endothelial monolayers treated with RSPO3 for 6 h. Green, phalloidin staining of actin fibers; blue, DAPI staining of nuclei. Microphotographs were taken using a Zeiss Axioskope equipped with Axio-CamMRm and AxioVision Rel.4.6 software and are representative of three independent experiments run in triplicates. Original magnification, 630×. (PDF 4093 kb)

Additional file 6: **Figure S5.** β-catenin alignment at adherens junctions in vascular endothelial monolayers treated with RSPO3. Immunofluorescence staining of β-catenin (red) and DAPI staining of nuclei (blue) in HCAEC either untreated, or treated with RSPO3 for 6 h. Microphotographs were taken using a Zeiss Axioskope equipped with Axio-CamMRm and AxioVision Rel.4.6 software and are representative of three independent experiments run in triplicates. Original magnification, 630×. (PDF 5145 kb)

Additional file 7: **Figure S6.** Induction of RSPO3 expression by IL-1β in human vascular endothelium. (A) Immunoblot of RSPO3 (31 kDa) in HCAEC treated with IL-1β for 24 h. In-gel stained 75 kDa band served as loading control and for immunoblot normalization in densitometric analysis. (B) RSPO3 expression levels quantified by densitometry analysis. Data are mean ± S.E.M of three independent experiments, **P < 0.01 versus untreated (none). (PDF 191 kb)

## Abbreviations

ECIS: Electric cell-substrate impedance sensing
HBMVEC: Human brain microvascular endothelial cells
HCAEC: Human coronary artery endothelial cells
HCMVEC: Human cardiac microvascular endothelial cells
HDMVEC: Human dermal microvascular endothelial cells
HPAEC: Human pulmonary artery endothelial cells
RSPO: R-spondin
