# Supplementary material for: RSPO3 impairs barrier function of human vascular endothelial monolayers and synergizes with pro-inflammatory IL-1
Source: Mol Med. 2018 Aug 29;24:45. doi: 10.1186/s10020-018-0048-z (PMC6116367; doi:10.1186/s10020-018-0048-z)
Supplement: Supplementary file 1 — Supplementary methods. (PDF 124 kb) [file 10020_2018_48_MOESM1_ESM.pdf]

Additional file 1

Supplementary methods

Cell culture

HCAEC (Cat. No. CC-2585), HPAEC (Cat. No. CC-2530), HCMVEC (Cat. No. CC-7030), HDMVEC (CC-2543, all from Clonetics, Lonza), and HBMVEC (Cat. No. ACBRI 376, Cell Systems) were propagated in EBM-2 medium (Clonetics, Lonza) supplemented with EGM-2MV Single Quots with 5% FBS (Clonetics, Lonza) in a Class 100 HEPA air filtered system (SteriCult, Fisher Scientific, Switzerland) as described [1]. All the five endothelial cell types used in this study were tested positively and functionally for CD31/105, von Willebrand Factor VIII, and acetylated low density lipoprotein uptake as guaranteed by the manufacturer (Lonza, Cell Systems). To avoid masked low-level contamination in cultures, culture medium devoid of antibiotics was used throughout the study. Only cells from passages 3- 6 were used for the experiments. Endothelial monolayers were treated with recombinant human RSPO3 (500 ng/mL, Cat. No. 120-44, PeproTech) and recombinant human IL-1β (20 U/mL, Cat. No. 200-01B, PeproTech). A dose response assessment performed with three different concentrations of RSPO3 (250 ng/mL, 500 ng/mL and 1000 ng/mL) revealed 500 ng/mL as the optimal dose required to produce significant reproducible changes in monolayer permeability (Additional file 3: Fig. S2). Therefore, 500 ng/mL of RSPO3 was chosen in all further experiments in this study. For all pipetting steps, TipOne aerosol barrier sterile filter pipet tips (USA Scientific) were used.

Immunoblotting

Immunoblotting of RSPO3 and quantification of band densities were carried out as described [2] with modifications. After resolving equal amounts of protein on Criterion Stain-Free Precast Gels (Cat. 567–8074, Bio-Rad) and transferring onto PVDF membranes, the membranes were incubated with blocking solution (5% BSA in 1X TBS containing 0.1%

Tween-20) for 1 h at room temperature (RT). The membranes were then washed thrice in 1X TBS containing 0.1% Tween-20 (TBST) and incubated with primary antibodies (diluted in blocking solution) at 4°C overnight. The following antibody was used with dilution indicated: rabbit anti-RSPO3, polyclonal (1:500, Cat. No. ab171010, Abcam). After washing thrice in 1X TBST, blots were incubated with anti-rabbit IgG- HRP-linked whole antibody (1:5000, Cat. No. NA934V, GE Healthcare UK Limited) for 1 h at RT. The blots were washed thrice in 1X TBST and visualized with ECL Plus Western blotting detection reagents (Amersham Bioscience) in a ChemiDoc MP imaging system (Bio-Rad). Densitometry analysis of blots were performed using Bio-Rad Image lab 5.2.1 software. In-gel stained total protein served as loading control for RSPO3 protein quantification.

Live recording of endothelial monolayer barrier function using ECIS

The response of the endothelial monolayer barrier to a particular stimulus is functionally assessed in real-time in a fully standardized manner by continuously recording changes in trans-endothelial electrical resistance (TEER) using ECIS [3]. Endothelial barrier function was continuously recorded using the 8W10E+ electrode chamber arrays and ECIS Z Theta system (Applied Biophysics) with associated software v.1.2.126 PC, as described [1]. Briefly, 8W10E+ arrays were coated with rat tail collagen type I (0.01% in sterile pyrogen-free water; BD Biosciences) for 12 h and the array electrodes were stabilized as per manufacturer’s instructions. A monodisperse suspension of human vascular endothelial cells in EGM-2 MV medium (Clonetics, Lonza) supplemented with 5% FBS was added to the arrays at a density of 80,000–90,000 cells/well and incubated up to 20- 24 h to form a uniform dense monolayer (Additional file 2: Fig. S1). Treatments were carried out adding 200 μL fresh medium with the respective stimuli into wells containing 200 μL of culture medium, yielding the final concentrations indicated in a total volume of 400 μL/well. Resistance of endothelial monolayer was continuously measured in Ohms at multiple

frequencies ranging from 62.6 Hz to 64 kHz. Each of the eight wells of the 8W10E+ arrays contain 40 electrodes that trace the monolayer at 40 different locations in every well. The measurements from each well were averaged. Resistance measurements at 4000 Hz indicative of cell-cell adhesion tightness are shown.

Immunofluorescence staining

Cells were grown up to a confluent monolayer stage in rat tail collagen type I coated four-chamber glass culture slides (Falcon), incubated with the indicated stimuli for 6 h and fixed immediately with 4% formalin buffer for 10 min. Cells were washed thrice in PBS, incubated with blocking solution (10% goat serum and 1% BSA in PBS) for 1 h at RT and immunofluorescence staining for β-catenin and VE-cadherin were performed as described [1, 4]. Rabbit -anti- β-catenin (diluted 1:150, Cat. No. 9562, Cell Signalling Technology) and rabbit-anti-VE-cadherin (diluted 1:100, Cat. No. 2158, Cell Signalling Technology) were used as the primary antibodies and detected by the Alexa 568 labelled goat anti-rabbit secondary antibody (1:2000, Cat. No. A11036, Molecular Probes, Invitrogen). The antibodies were diluted in PBS containing 1% goat serum and 0.1% BSA. To stain for cellular actin, formalin fixed cells were incubated with Alexa Fluor 488 Phalloidin (diluted 1:80, Molecular Probes, Invitrogen, USA) for 1 h at RT in the dark. Quantitative assessment of gap index and gap size index were carried out as described [5]. Gaps were quantified from 8 fields (≥ 75% cells/field) of VE-cadherin immunostained endothelial monolayers from each of the two experimental groups (nontreated, RSPO3 treated). Total cell area and inter-endothelial gap area were quantified using ImageJ based Fiji software.

References

1. Skaria T, Bachli E, Schoedon G. Wnt5A/Ryk signaling critically affects barrier function in human vascular endothelial cells. Cell Adhesion & Migration. 2017; **11:**24-38.
2. Skaria T, Bachli E, Schoedon G. WIF1 prevents Wnt5A mediated LIMK/CFL phosphorylation and adherens junction disruption in human vascular endothelial cells. J Inflamm (Lond). 2017; **14:**10.
3. Bernas MJ, Cardoso FL, Daley SK, Weinand ME, Campos AR, Ferreira AJG, Hoying JB, Witte MH, Brites D, Persidsky Y, et al. Establishment of primary cultures of human brain microvascular endothelial cells to provide an in vitro cellular model of the blood-brain barrier. Nat Protocols. 2010; **5:**1265-1272.
4. Skaria T, Burgener J, Bachli E, Schoedon G. IL-4 Causes Hyperpermeability of Vascular Endothelial Cells through Wnt5A Signaling. PLOS ONE. 2016; **11:**e0156002.
5. Fraccaroli A, Pitter B, Taha AA, Seebach J, Huveneers S, Kirsch J, Casaroli-Marano RP, Zahler S, Pohl U, Gerhardt H, et al. Endothelial alpha-parvin controls integrity of developing vasculature and is required for maintenance of cell-cell junctions. Circ Res. 2015; **117:**29-40.
