# Supplementary figures and images for: RSPO3 impairs barrier function of human vascular endothelial monolayers and synergizes with pro-inflammatory IL-1
Source: Mol Med. 2018 Aug 29;24:45. doi: 10.1186/s10020-018-0048-z (PMC6116367; doi:10.1186/s10020-018-0048-z)

**A**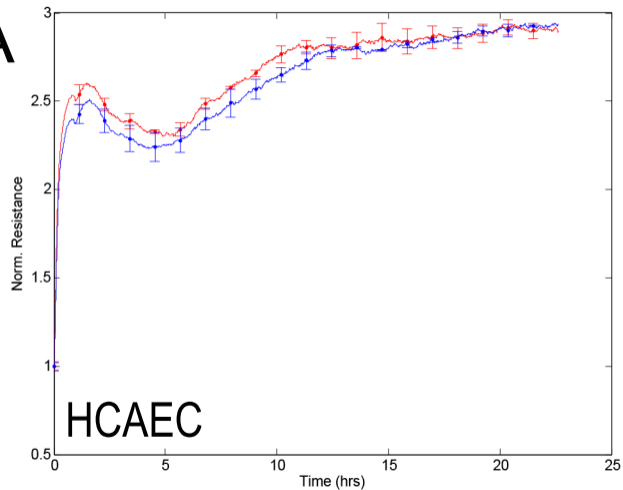**B**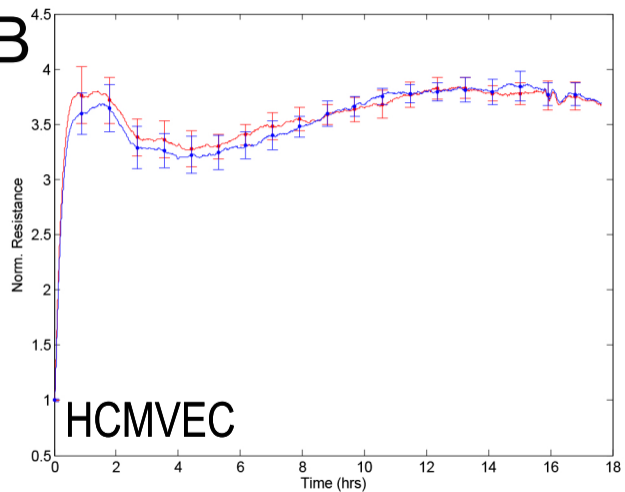

Supplement: Supplementary file 2 — Figure S1. Confluent tight endothelial monolayer formation in 8W10E+ ECIS arrays. Immediately after cell seeding, resistance measurements (in Ohms) were started and are displayed as normalized resistance (subsequent values were divided by initial values). Increase in resistance with respect to time denotes that cells were forming contacts between each other. The steady state shows the stage at which maximum resistance is reached to form a tight monolayer. Resistance measurements were carried out in duplicate wells which were grouped and averaged to plot as single curve. Error bars represent mean ± S.E.M. Figures show the original plot of resistance measured at 4000 Hz (indicative of cell-cell adhesion tightness). Red, blue: record of monolayer formation of untreated (A) HCAEC and (B) HCMVEC grown in duplicate wells from two independent experiments. All treatments for subsequent assessment of barrier function were started after formation of stable tight monolayers. (PDF 636 kb) [file 10020_2018_48_MOESM2_ESM.pdf]

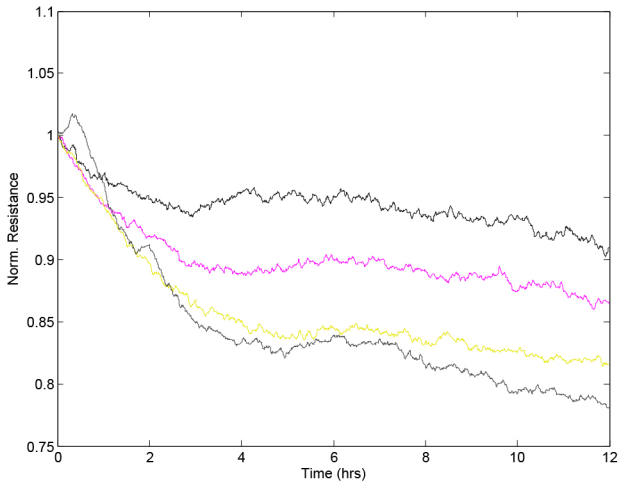

Supplement: Supplementary file 3 — Figure S2. Dose response assessment of RSPO3 on endothelial barrier function using the ECIS system. Uniform tight monolayers of HCAEC cultured in stabilized and collagen coated 8W10E+ ECIS array chambers were treated with 250 ng/mL, 500 ng/mL and 1000 ng/mL RSPO3. Data shown are the original resistance measurements conducted at 4000 Hz (indicative of cell-cell adhesion tightness) and are representative of three independent experiments. Black, vehicle; Purple, RSPO3 (250 ng/mL); Yellow, RSPO3 (500 ng/mL); Grey, RSPO3 (1000 ng/mL). (PDF 304 kb) [file 10020_2018_48_MOESM3_ESM.pdf]

**A**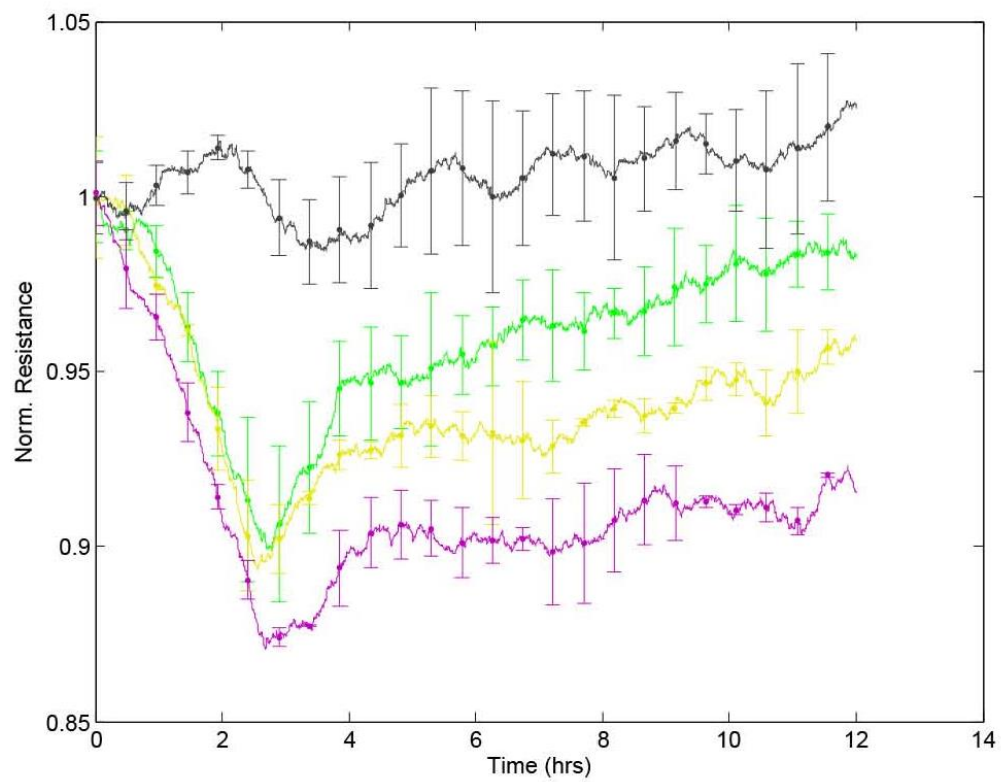**B**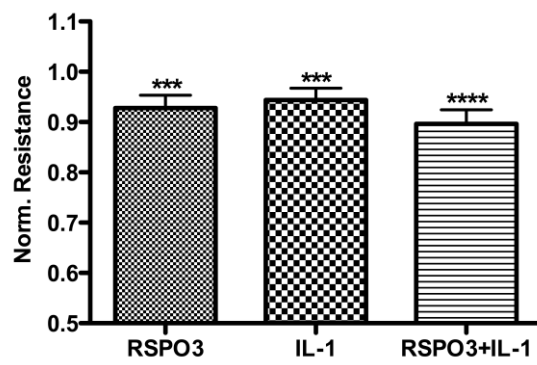

Supplement: Supplementary file 4 — Figure S3. Assessment of barrier function in dermal microvascular endothelial monolayers. Uniform tight monolayers of HDMVEC cultured in stabilized and collagen coated 8W10E+ array chambers were treated with RSPO3, IL-1β or a combination of RSPO3 and IL-1β. (A) Original ECIS plot of resistance measurements (line graph) from one representative of three independent experiments run in duplicates at 4000 Hz (indicative of cell-cell adhesion tightness). (B) Bar graph showing the data of barrier function measurements continuously recorded for and at 12 h from three independent experiments run in duplicates. Black, vehicle; Green, IL-1β; Yellow, RSPO3; Purple, RSPO3 + IL-1β. Error bars are mean ± S.D. ***P < 0.001 versus vehicle treatment, ****P < 0.001 versus RSPO3 and IL-1β single treatments. (PDF 242 kb) [file 10020_2018_48_MOESM4_ESM.pdf]

none

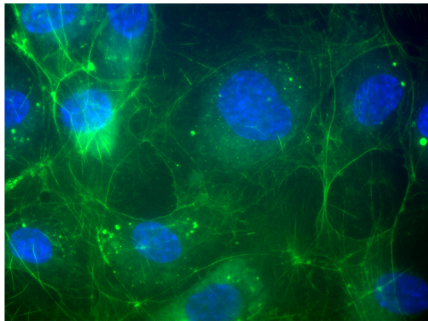

RSP03

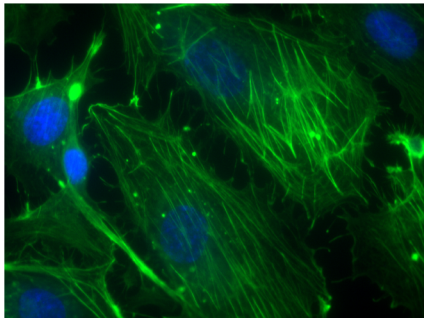

Supplement: Supplementary file 5 — Figure S4. Actin cytoskeletal changes in vascular endothelial monolayers treated with RSPO3 for 6 h. Green, phalloidin staining of actin fibers; blue, DAPI staining of nuclei. Microphotographs were taken using a Zeiss Axioskope equipped with Axio-CamMRm and AxioVision Rel.4.6 software and are representative of three independent experiments run in triplicates. Original magnification, 630×. (PDF 4093 kb) [file 10020_2018_48_MOESM5_ESM.pdf]

none

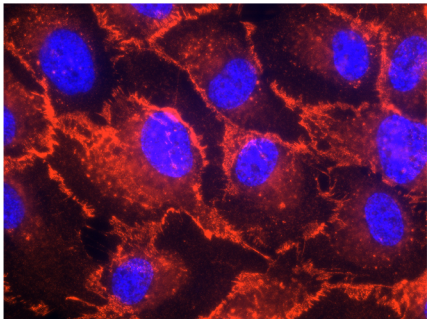

RSP03

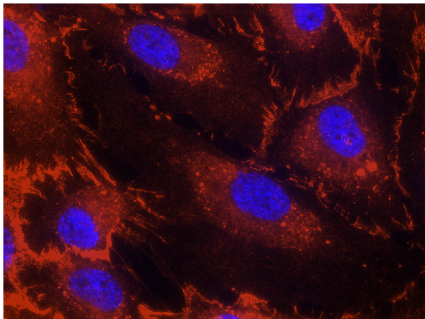

Supplement: Supplementary file 6 — Figure S5. β-catenin alignment at adherens junctions in vascular endothelial monolayers treated with RSPO3. Immunofluorescence staining of β-catenin (red) and DAPI staining of nuclei (blue) in HCAEC either untreated, or treated with RSPO3 for 6 h. Microphotographs were taken using a Zeiss Axioskope equipped with Axio-CamMRm and AxioVision Rel.4.6 software and are representative of three independent experiments run in triplicates. Original magnification, 630×. (PDF 5145 kb) [file 10020_2018_48_MOESM6_ESM.pdf]

**A**

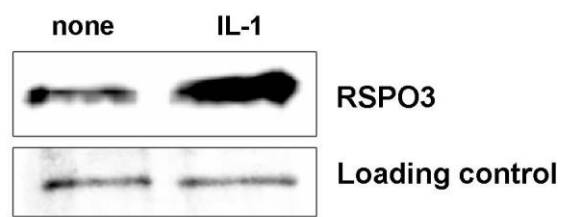

**B**

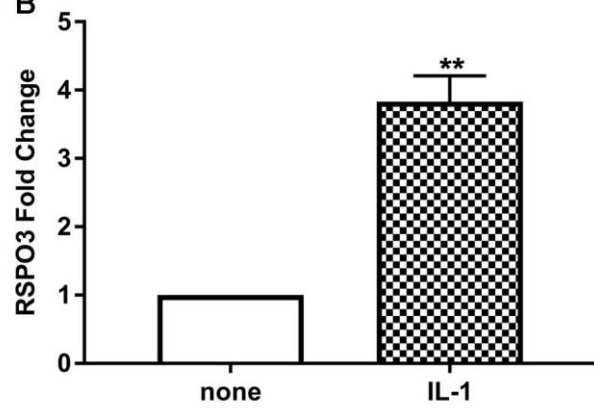

Supplement: Supplementary file 7 — Figure S6. Induction of RSPO3 expression by IL-1β in human vascular endothelium. (A) Immunoblot of RSPO3 (31 kDa) in HCAEC treated with IL-1β for 24 h. In-gel stained 75 kDa band served as loading control and for immunoblot normalization in densitometric analysis. (B) RSPO3 expression levels quantified by densitometry analysis. Data are mean ± S.E.M of three independent experiments, **P < 0.01 versus untreated (none). (PDF 191 kb) [file 10020_2018_48_MOESM7_ESM.pdf]
